# The optimal interval before receiving SARS-COV-2 vaccination for patients who have received Anti-CD 20 monoclonal antibodies

**DOI:** 10.1080/21505594.2022.2146380

**Published:** 2022-11-16

**Authors:** Kexin Liu, Jinyu Li, Gaosi Xu

**Affiliations:** a Department of Nephrology, The Second Affiliated Hospital of Nanchang University, Nanchang, China; b Grade 2019, The Second Clinical Medical College of Nanchang University, Nanchang, Jiangxi, China; c Grade 2020, The First Clinical Medical College of Nanchang University, Nanchang, Jiangxi, China

**Keywords:** Anti-cd20, vaccine, rituximab, Sars-CoV-2, COVID-19

## Abstract

The optimal interval before receiving SARS-COV-2 vaccination for patients who have received anti-CD 20 monoclonal antibodies remains unclear. We considered original studies up to 29 October 2022 and conducted searches in Embase,Medrxiv, PubMed, and SSRN. We excluded search results that did not match our research question’s subject. Human immune response outcomes were analysed inpatients who had previously received anti-CD20 antibody therapy. We analyzed the collected results using sensitivity curves and forest plots. Twenty-eight studies with a total of 1455 subjects receiving anti-CD20 monoclonal antibodies were included in the present analysis. The humoral immune response rates to the time between the last anti-CD20 treatment and vaccination for 3–6 months, 6 months,6–9 months, and 9–12 months were 0.23 (95% CI 0.14 to 0.36), 0.36 (95% CI 0.19 to 0.58), 0.49 (95% CI 0.35 to 0.64) and 0.64 (95% CI 0.48 to 0.77),respectively. The humoral immune response rates were.16 (95% CI 0.03 to 0.57) when B cell was 0/ul, and 0.49 (95% CI 0.38 to 0.61)when B cells were more than 5/ul. The humoral immune response rate for multiple sclerosis was 0.39 (95% CI 0.22 to 0.60) and 0.48 (95% CI 0.29 to 0.68) for B-cell non-Hodgkin lymphoma. The area underneath the curve(AUC) was 0.69 with a cut-off value of 5.5 months. The present results suggested that the optimal interval for SARS-COV-2 vaccination after the final dose of anti-CD20 monoclonal antibody was 5.5 months.

## Introduction

According to the World Health Organization, the COVID-19 pandemic, which began at the beginning of 2019, will cause 6.5 million fatalities and 624 million confirmed cases worldwide by 23 October 2022. We are still fighting against it, and there are still many unknowns. Fortunately, we have developed the corresponding vaccine, and with the SARS-CoV-2 vaccine’s broad use in numerous nations, it has played a preventive role. However, for some special populations, the patients receiving anti-CD20 treatment, the vaccination’s impact on their humoral and cellular immunity is still unknown.

The response to immunization in patients receiving active anti-CD20 medication is reported to be modest and close to 0%, but it appears to gradually improve over time [1]. Regardless of the illness or other treatments, numerous studies have now demonstrated decreased antibody responses and seroconversion rates for anti-CD20 treated patients following COVID-19 vaccination [2–5]. Although anti-CD20 therapy impairs humoral responses, patients can develop strong T-cell responses to the COVID-19 mRNA vaccine, which may be crucial in reducing the consequences of severe COVID-19 [6]. Baker’s article mentions that both T cell-dependent and independent responses were significantly impaired in patients for at least six months following anti-CD20 medication therapy [7]. According to the current expert agreement, patients who have been receiving anti-CD20 medication should wait at least 6 months before receiving vaccination after weighing the risks and benefits of anti-CD20 medicine injections. However, there is no higher level of medical evidence.

Therefore, due to the lack of studies on the cellular and humoral responses of anti-CD20-treated patients, our present study revolves around the analysis of anti-CD20-treated patients receiving the vaccine and gives a higher level of evidence-based medical evidence on whether 6 months is the cut-off point for anti-CD20 treated patients waiting for vaccination.

## Methods

We carried out a systematic review and meta-analysis of preprints and peer-reviewed articles that were accessible online, and we presented our findings in accordance with the Preferred Reporting Items for Systematic Reviews and Meta-Analyses.

### Eligibility criteria

We saw as possibly eligible for inclusion any original research papers that examined the serological and/or cell-mediated reactions to SARS-CoV-2 vaccination in patients receiving anti-CD20 treatment. Unspecified periods between vaccination and blood sampling, unspecified methodology for detecting antibody-mediated or cell-mediated immunity (specification of manufacturers and detection kits mandatory), lower or equal to the three investigated participants, and missing numbers of positive versus negative humoral or cell-mediated immunity were among the prespecified exclusion criteria. Additionally, any search results that did not pertain to the subject of our study question were removed, as well as review and guideline papers.

### Information sources and search strategy

Online searches were performed using title/abstract without regard to language using PubMed (up to 29 October 2022), Embase (up to 29 October 2022), preprint services medrxiv, SSRN, and SSRN-Lancet (1 January 2020–29 October 2022).

A PubMed title/abstract search for “rituximab OR anti-cd20 AND vaccination AND covid” was conducted. Embase’s title/abstract section was searched for “rituximab OR anti-cd20 AND vaccination AND covid.” Medrxiv was searched for “Rituximab AND Covid AND Vaccine.” Furthermore, the term “anti-CD20 AND covid AND vaccination” was researched. A third search was carried out using the keyword “anti-CD20 AND covid AND vaccination.” SSRN was searched for “Rituximab AND Covid AND Vaccine.” Another search was performed with the phrase “anti-CD20 AND covid AND vaccination.” The phrase “rituximab AND covid AND vaccination” was found in SSRN Preprints by The Lancet. Another search was conducted using the term “anti-CD20 AND covid AND vaccination.”

### Selection process

We followed the Cochrane recommendations when we carried out the study selection process. In addition to two writers (KL and GX) individually evaluating all search results from the EMBASE database and preprint sites, two authors (KL and JL) independently evaluated all search results from the PubMed database. When there were disagreements over the choices, a third author (JL or GX, as appropriate) was consulted. Each of the three authors went over the inclusion and exclusion criteria again, and they then discussed and double-checked their decisions. In every instance, disagreements might be conclusively settled by the unanimous consent of all authors. Due to ongoing conflicts, we did not need to use the predetermined form of majority decisions. We did not use any automated tools.

### Data collection process

Each reviewer carefully copied and independently downloaded the tabular and text data of study population subsets having a history of anti-CD20 treatment. In some circumstances, image analysis was used to extract data from graphical figures. We did not use any automated tools.

### Data items

Along with the previously mentioned inclusion and exclusion criteria, we gleaned the following data from the studies we searched.

#### Primary outcome data

A percentage of patients who received anti-CD20 therapy had no prior COVID-19 disease history, finished the SARS-CoV-2 immunization series, and had positive serological results.

#### Data for subgroup generation

Primary outcome data are broken down by the amount of time since the previous anti-CD20 therapy (3–6 months, 6 months, 6–9 months, and 9–12 months), B cell depletion counts (0/ul, 0-5/ul, >5/ul), the dose of injected anti-CD20 drugs and disease types of the study population.

#### Data for quality evaluation

Research design, cell-mediated immune (CMI) response measurement technique, detection kit manufacturers, and corresponding cut-off values for test positive, study title, digital object identifier, and PubMed identifier are all required for repeated duplicate checks. We obtained these data from the manufacturer’s websites in cases where cut-off values of a manufacturer’s kit for antibody or CMI response testing were not mentioned in the procedures section.

### Risk of bias assessment

Using the Newcastle- Ottawa Scale for evaluating the quality of non-randomized studies in meta-analyses by Wells et al, we manually evaluated the risk of bias in the included studies. For each study, three researchers (KL, JL, and GX) independently assigned a range of quality criteria (minimum 0, maximum 9). Results were summarized by a fourth researcher (JL) using a predetermined mode of majority decision. It was anticipated that at least 4 weeks after the second immunization was finished was the cut-off for an ideal follow-up time.

### Effect measures

The number and percentage of respondents were used for the synthesis and presentation of results for humoral immunity outcomes.

### Synthesis methods

Using Microsoft Excel to tabulate the research and compare them to a list of exclusion criteria, a preliminary synthesis was created. In the studies that were included, there were no missing data. Using the Der Simonian and Laird approach, we conducted a random-effects meta-analysis of proportions, stabilizing the variances by computing pooled estimates using the Freeman-Tukey Double Arcsine Transformation. The inverse-variance fixed-effect model’s I^2^ metric was used to quantify statistical heterogeneity. The Wilson score with 95% CI for each study is shown. We carried out the previously mentioned prespecified subgroup analysis to investigate potential reasons for study heterogeneity. Revman 5.4.1 performed quantitative analysis and produced graphical displays. The results are converted by the formula: P=OR/(1+OR). No sensitivity analyses were performed. ROC curves are plotted using Graphpad Prism. ROC curve data collection using Microsoft Excel, Jorden index calculation done through Microsoft Excel, and determination of cut-off values.

### Reporting bias assessment

A funnel plot and a regression test for funnel plot asymmetry were used to evaluate the effects of small studies.

### Certainty assessment

No processes were used to evaluate the body of evidence’s credibility.

## Results

### Search results

**Figure 1.**
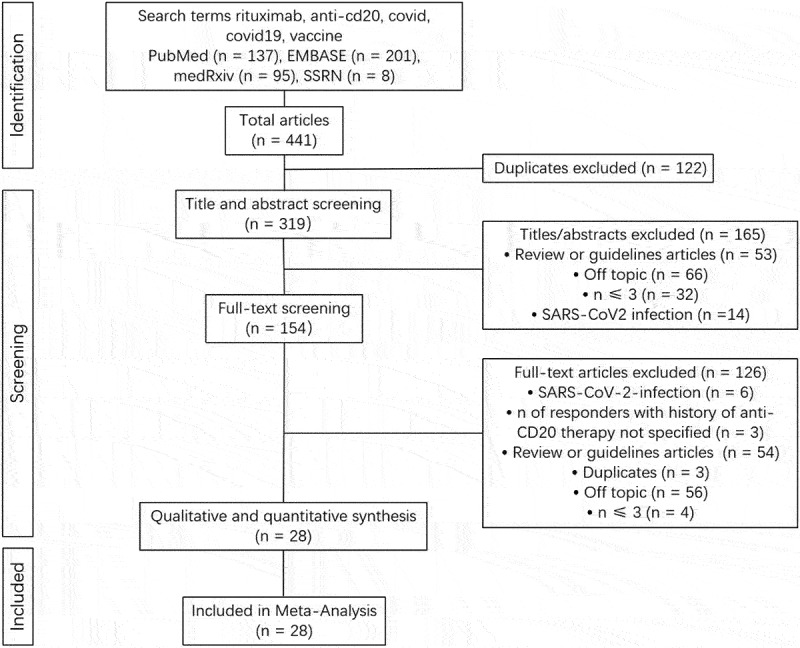
Flow chart describing the study search and selection process.

Figure 1 presents the study selection procedure in detail. The results of searches in Embase (12) and PubMed (12) were 137 and 201, respectively. Searches on preprint sites turned up 95 papers from medrxiv and 8 studies from SSRN and SSRN Preprints with The Lancet. After 122 duplicate studies were removed for eligibility evaluation, 319 studies remained. The screening of titles and abstracts resulted in the exclusion of 165 additional articles. The subsequent exclusion of 126 papers followed the full-text screening of the remaining 154 articles (Supplementary Table 3). Twenty-eight studies totaling 1455 participants had data that fully complied with the inclusion criteria, therefore they were eventually included in the meta-analysis.

### Study characteristics

Table 1 summarizes the study characteristics of the included publications [4,6,8–33]. Data details of the roc curve are shown in the appendix.

**Table 1.** List of the included studies.

Author	Time since the final anti-CD20 therapy	Vaccine	No. of humoral analysis	% responders	B cell counts	Population(disease)
Avivi et al [8]		BP	42	31.0	4.8/ul	DLBCL,B-NHL(FL,MZL,MCL)
	≤6 months		19	10.5		
	>6 months		23	47.8		
Bitoun et al [9]	≤6 months	BP	24	29.2	NA	AID
Bonelli et al [10]	6 months	BP,M,mRNA,CV	55	27.3	NA	Arthritis,Connective tissue diseases,Vasulitis,MS,IgG4-related disease
Brill et al [11]		BP	27	51.9	NA	MS
	<5 months		17	35.5		
	≥5 months		9	88.9		
Buchwinkler et al [12]	8 months	BP,mRNA	30	46.7	NA	CKD
Carruthers et al [13]	≤12 months	AZ,BP	15	77.8	NA	AAV
Cohen et al [14]	3 months	BP	34	8.8	NA	VAHL(lymphoma,myeloma)
Diefenbach et al [15]	42d	BP, M	24	25	NA	NHL,CLL,SLL,DLBCL,FL,MCL,MZL,Low-grade B cell lymphoma,BL
Firinu et al [16]	5.5 months	BP	7	28.6	NA	IMIDs(RA,SLE,MSD)
Gadani et al [17]	30d	JJ,M,BP,unsure	39	56.4	NA	MS
Gurion et al [18]	0-45d	BP	34	2.9	NA	Lymphomas
	46-120d		21	23.8		
	121-180d		4	25		
	181-365d		7	12.3		
	>366d		21	81.0		
Haidar et al [19]	≤12 months	BP,M,mRNA,CV,JJ	51	17.7	NA	IDD
Herishanu et al [4]	5 months	BP	22	50	NA	CLL
	>12 months		55	94.1		
Jinich et al [20]		BP.M	56	51.8	>5ul	RA,SLE,Sjögren’s syndrome,SSc,PMR,IgG4-related disease,ANCA-associated vasculitis
				48.2	0/ul	
	<6 months		21	14.9		
	6~12 months		11	45.5		
	>12 months		24	87.5		
König et al [21]	4 months	BP	183	20.2	NA	MS
Kornek et al [22]	6 months	BP,M	82	33	NA	MS
Kornek et al [23]	6 months	M	82	69.5	NA	MS
Krasselt et al [24]	215d	JJ,M,BP,AZ	29	10.3	0/ul	autoimmune inflammatory rheumatic diseases
Lim et al [25]	6 months	BP, CV	31	45.2	1/ul	HL
	7–12 months		4	75		
	>12 months			100		
Madelon et al [6]	24.9 weeks	BP, M	26	3.8	0/ul	MS, RD
Marty et al [26]		BP,M,JJ	19	94.7		AVV
			11	90.9	<1/ul	
			8	100.0	≥1/ul	
Moor et al [27]	≤6 months	BP, M	96	49.0	NA	autoimmune disease, malignant tumour, renal transplantation
	6~12 months		96	67.7		
Novak et al [28]	15.1 weeks	BP	60	13.5	NA	MS
Perry et al [29]	>6 months	BP	66	66.7	NA	B-NHL
	≤6 months		55	7.3		
	>9 months		51	84.3		
	≤9 months		70	7.1		
Schulz et al [30]	166 days	M	120	67	NA	Hematological malignances
Stefanski et al [31]	9 months	BP,M,CV	19	42.1	10.0/ul	RA, AVV
Tanguay et al [32]		BP, M	55	45.5	0~5/ul	DLBCL,B-NHL(FL,MZL,MCL,LPL,CLL)
	≤1 year		22	4.5		
	1~2 years		16	56.3		
	>2 years		17	88.2		
Tvito et al [33]	<6 months	BP	28	3.6	NA	NHL

AAV, antineutrophil cytoplasmic antibody; AID, autoimmune diseases; AZ, Astra-Zeneca; B-NHL, B-cell non-Hodgkin lymphoma; BL, Burkitt lymphoma; BP, BioNTech-Pfizer; CLL Chronic Lymphocytic leukaemia; DLBCL, diffuse large cell B-cell lymphoma; CV, CoronaVac; FL, follicular lymphoma; HL, Hodgkin lymphoma; LBCL, Low-grade B cell lymphoma; IDD, immune deficiency disorders; IMIDs, immune-mediated inflammatory diseases; JJ, Johnson-Johnson; M, Moderna; MCL, mantle cell lymphoma; MS, Multiple Sclerosis; MSD, miscellaneous systemic disorders; MZL, marginal zone lymphoma; NHL, Non-Hodgkin lymphoma; RA, rheumatoid arthritis, SLE, systemic lupus erythematosus; VAHL, hypermetabolic lymphadenopathy.

### Risk of bias

Supplementary Table 4 reveals the findings of the risk of bias analysis for each of the listed research. Applying the NOS, eight of the nine quality rating criteria could be satisfied (the ninth, “Adequacy of follow-up,” was not relevant). The 28 included studies have a low to moderate risk of bias ratings.

### Results of individual studies

The Methods section provides specifics on the proportion calculations for meta-analysis utilizing a random-effects model computation of the effect size. The log [odds ratio] and Standard Error (SE) of each study are first shown separately. The penultimate column, with a 95% confidence interval, displays the percentage weight of the particular study. The final column displays the combined odds ratio estimates.

### Humoral response to vaccination

**Figure 2.**
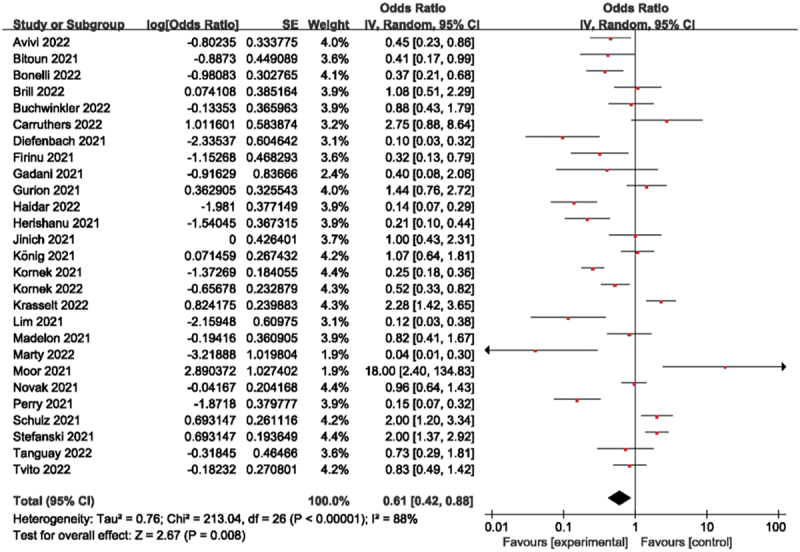
Humoral immune responses across all included studies.

With responder rates ranging from around 3.8% to nearly 94%, humoral responses were quite diverse, with a 38% overall humoral immune response rate. Heterogeneity was demonstrated in this study collection by an I^2^ of 88% (Figure 2). Studies were therefore submitted to predetermined subgroup analyses, which included the length of time since the last anti-CD20 medication and the indication for anti-CD20 treatment.

**Figure 3.**
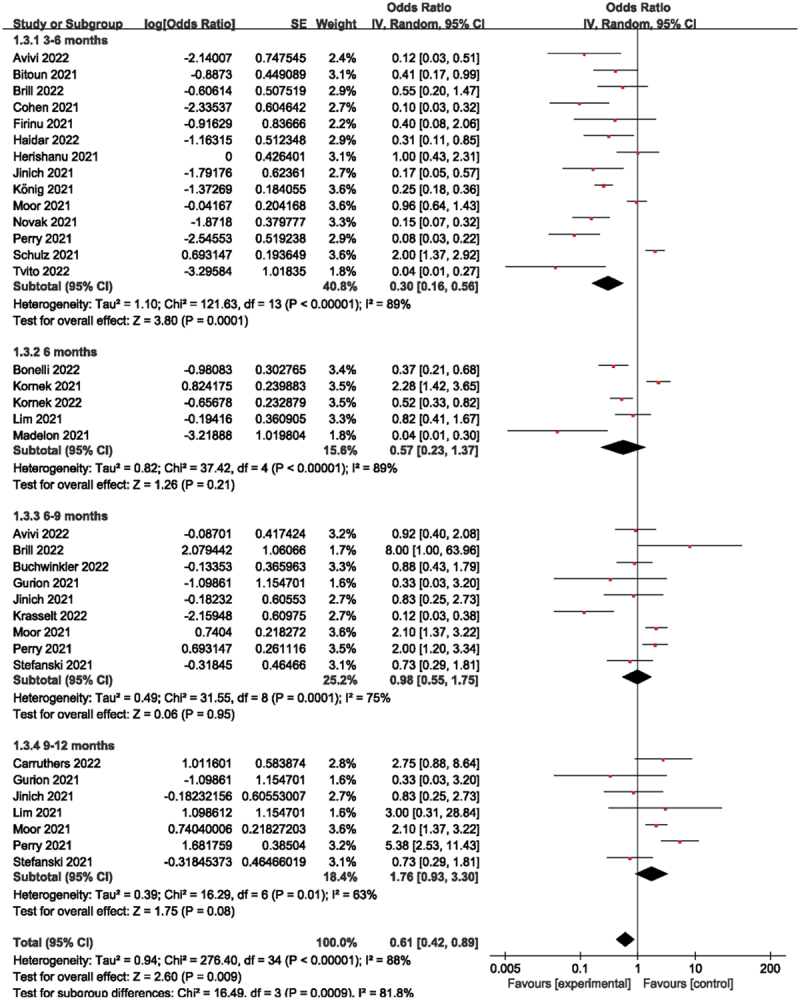
Humoral immune responses according to prespecified subgroups of 3-6, 6, 6–9 and 9–12 months since the last dose of anti- CD20 therapy.

Stratified as 3–6 months, 6 months, 6–9 months, and 9–12 months depending on how long it had been after the last anti-CD20 medication and immunization (where the 6 months interval was taken as 1 month and the other intervals as 3 months), the forest plots showed that the humoral immune response rates corresponded to 23%, 36%, 49%, and 64% for 3–6 months, 6 months, 6–9 months, and 9–12 months, respectively, the overall humoral immune response rate was 38% (Figure 3). Studies with shorter intervals reported significantly fewer responders, such as the humoral immune response rate from 3–6 months compared to 9–12 months (23% vs 64%). Of these, when the time interval was 6 months, in contrast to other short-term studies, Kornek et al [23] found a humoral immune response rate of 70%, which may explain the considerable heterogeneity of this cohort.

**Figure 4.**
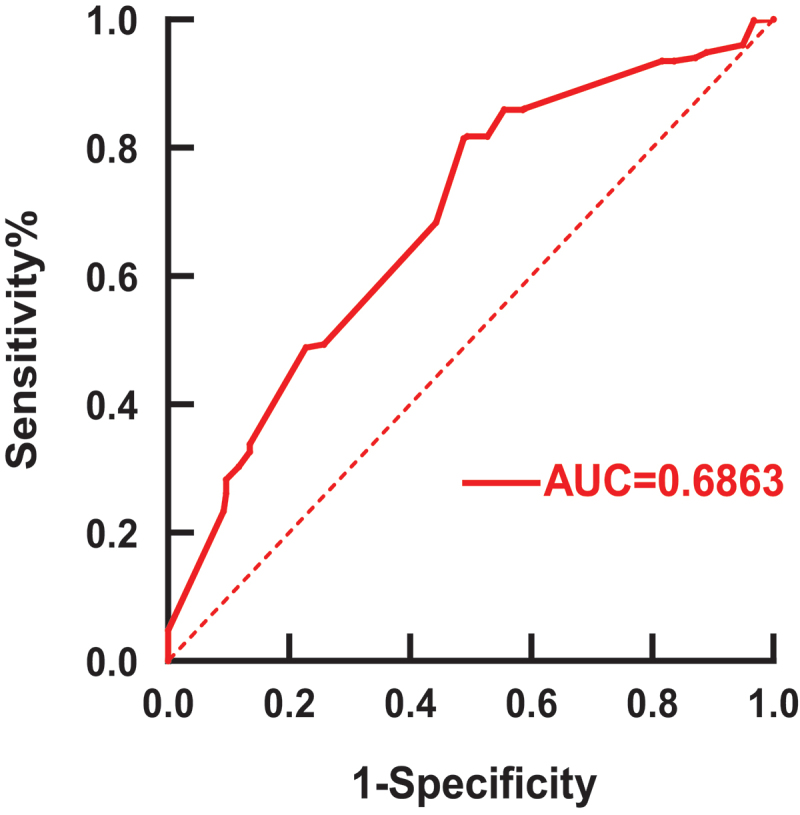
ROC curve on the time interval of SARS-CoV-2 vaccination in 1455 patients treated with anti-CD20.The vertical coordinates indicate that all patients who actually received the vaccine were greater than 5.5 months from the last dose of anti-CD20 monoclonal antibody therapy were not infected with the COVID-19. The abscissa indicates that all patients who actually received the vaccine were more than 5.5 months from the last dose of anti-CD20 monoclonal antibody therapy were infected with the COVID-19.

For the question of how long it has been since the last anti-CD20 treatment, ROC curves were created (Figure 4). From the sensitivity curve, the area underneath the curve (AUC) was 0.6863 with good confidence, and the cut-off value of 5.5 months was obtained by data processing through Microsoft Excel (Supplementary Table 2).

We explored whether current counts of B cell depletion are related to worse seroconversion rates, similar to the time window since the last anti-CD20 medication. Patients with low levels of B cells had fewer responders, The humoral immune response rate was 16% when B cells were 0/ul, and 49% when B cells were 0-5/ul, and >5/ul (Supplementary Figure 3).

Supplementary Figure 1 shows the humoral immune response rate for MS was smaller than for B-NHL (39% vs 48%), depending on the disease obtained. The overall humoral immune response rate for disease types was 42%. No results are available on the dose of CD20 due to the small amount of literature and the small sample.

### Sensitivity analyses and reporting bias

**Figure 5.**
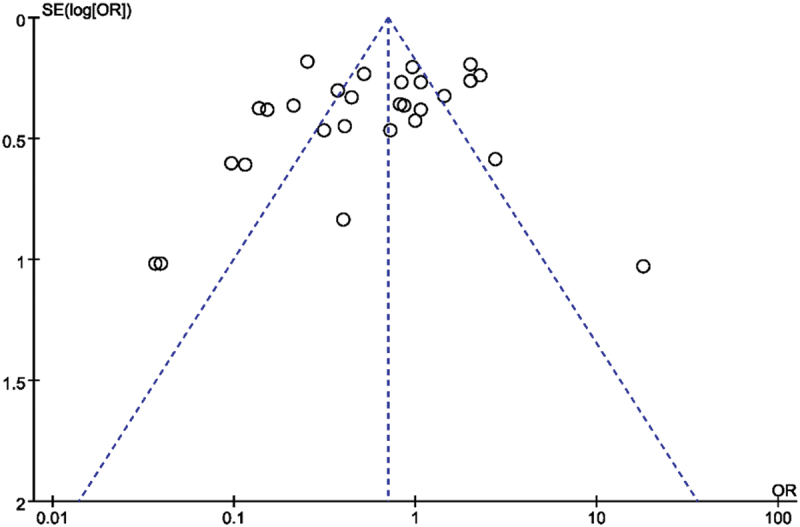
Funnel plot of all included studies.

No sensitivity studies were carried out. The Funnel plot showed no evidence of small-study effects (Figure 5).

## Discussion

The current work focuses on determining the optimal timing of the last anti-CD20 interval vaccination and outlines the rate of humoral immunity after SARS-CoV-2 immunization. The subgroup in this meta-analysis is the finest, and this meta-analysis currently has the biggest sample size of any analysis. These investigations were based on twenty-eight studies of patients who had previously received anti-CD20 therapy. This study was done because there is only expert consensus and no higher level of evidence from evidence-based medicine in determining the interval between the most recent anti-CD20 treatment and immunization in individuals who have received anti-CD20 treatment in the past. By performing ROC analysis on 1455 patients, we obtained a cut-off value of 5.5 (unit: months), which means that the optimal value for the interval between the most recent anti-CD20 treatment and immunization in individuals who have received anti-CD20 treatment is 5.5 months. This is consistent with the value taken for the time interval in Firinu et al [16], and Schulz et al [30].

In Firinu’s study, they analysed the vaccination response in 102 anti-CD20 patients. Compared to healthy controls (n = 551), all healthy controls showed serological conversion when the patient’s last anti-CD20 treatment was 1 month from vaccination, while 94% of patients did not respond, and when the patient’s last anti-CD20 treatment was 5 months from vaccination, there was a serological conversion in both patients and control groups, with no significant difference. Similarly, in Schulz’s study, naive B cells in immunocompromised haematological patients were measured to predict their humoral vaccination response. They performed anti-SARS-CoV-2 immunoassays at different intervals after the last B cell depletion treatment in 120 clinical immunocompromised hematological patients and concluded that it took at least 166 days between the last immunotherapy treatment and vaccination.

For the rate of humoral immunity following SARS-CoV-2 immunization, this meta-analysis collects recent months of the literature (up to 29 October 2022) and refines the interval between the final anti-CD20 medication and vaccination. Patients treated with the final dose of anti-CD20 monoclonal antibody were divided into positive and negative groups according to whether they were infected with COVID-19 after receiving the vaccine at different times. Data on the time it takes each group of patients to receive the vaccine were collected separately, data were processed, ROC curves were made, and cut-off values were determined. B-cell depletion was also graded, and the anti-CD20 dose used when treating patients with anti-CD20 was also collected.

The antibody titres of the SARS-CoV-2 vaccine were found to be significantly heterogeneous, and a subgroup analysis revealed that the duration since the last anti-CD20 treatment, the number of B cells, the dose of anti-CD20, and the disease type treated with anti-CD20 all had a significant impact on the humoral immune response rate.

By analysing Figure 3, we found that the humoral immune response rate increased with increasing time intervals, which may be connected to the gradual rise in B-cell counts following the cessation of anti-CD20 treatment, but also by the type of disease, type of vaccine used, or anti-CD20 dose, etc. These factors may contribute to the heterogeneity of this subgroup. In the 6-month subgroup analysis, it was found that Kornek et al [23] had a greater impact on this subgroup, so we decreased the heterogeneity of this subgroup by removing this literature in Revman 5.4.1 and then performing forest plot analysis and found that the heterogeneity I^2^ (89% vs 66%) and *P* value (0.21 vs 0.009) of this subgroup were significantly lower (Supplementary Figure 2). Comparing this article with the other articles in this subgroup, it was found that all the other articles used the BP vaccine, while this article used the M vaccine, which may be due to the different types of vaccines used, as well as the influence of region, population, and time.

There are certain restrictions on the evidence base used in this study. Due to the arbitrary scheduling of a literature search (29 October 2022) of quickly developing knowledge databases, the current review method was constrained, making the evidence tentative rather than conclusive and necessitating future meta-analyses. In addition, no unpublished research or clinical trial registry data were checked, and no other experts on the subject were contacted. Although this is reflected in a separate meta-analysis, several potential sources of variability in the seroconversion frequencies in the study population were excluded from the subgroup analysis and were not distinguished based on the various experimental methods of detecting blood antibodies. Finally, because population-level data were insufficient for differentiating across vaccination types, we did not examine the seroconversion rates of the various vaccines provided. When evaluating the evidence base further, analyses like these are required.

## Supplementary Material

Supplemental Material

## Data access statement

The authors confirm that the data supporting the findings of this study are available within the article and its supplementary materials.
