# Supplementary material for: The optimal interval before receiving SARS-COV-2 vaccination for patients who have received Anti-CD 20 monoclonal antibodies
Source: Virulence. 2022 Nov 16;13(1):2012–21. doi: 10.1080/21505594.2022.2146380 (PMC9673919; doi:10.1080/21505594.2022.2146380)
Supplement: Supplemental Material [file KVIR_A_2146380_SM2450.docx]

**The Optimal Interval before Receiving SARS-COV-2 Vaccination for Patients Who Have Received Anti-CD 20 Monoclonal Antibodies**

**Supplementary appendix**

**Table of contents**

**Supplementary Figure 1**

**Supplementary Figure 2**

**Supplementary Figure 3**

**Supplementary Table 1: Area under the ROC curve**

**Supplementary Table 2: Sensitivity, specificity, and Jorden index of ROC curve**

**Supplementary Table 3: List of screened studies**

**Supplementary Table 4: Risk of bias analysis**


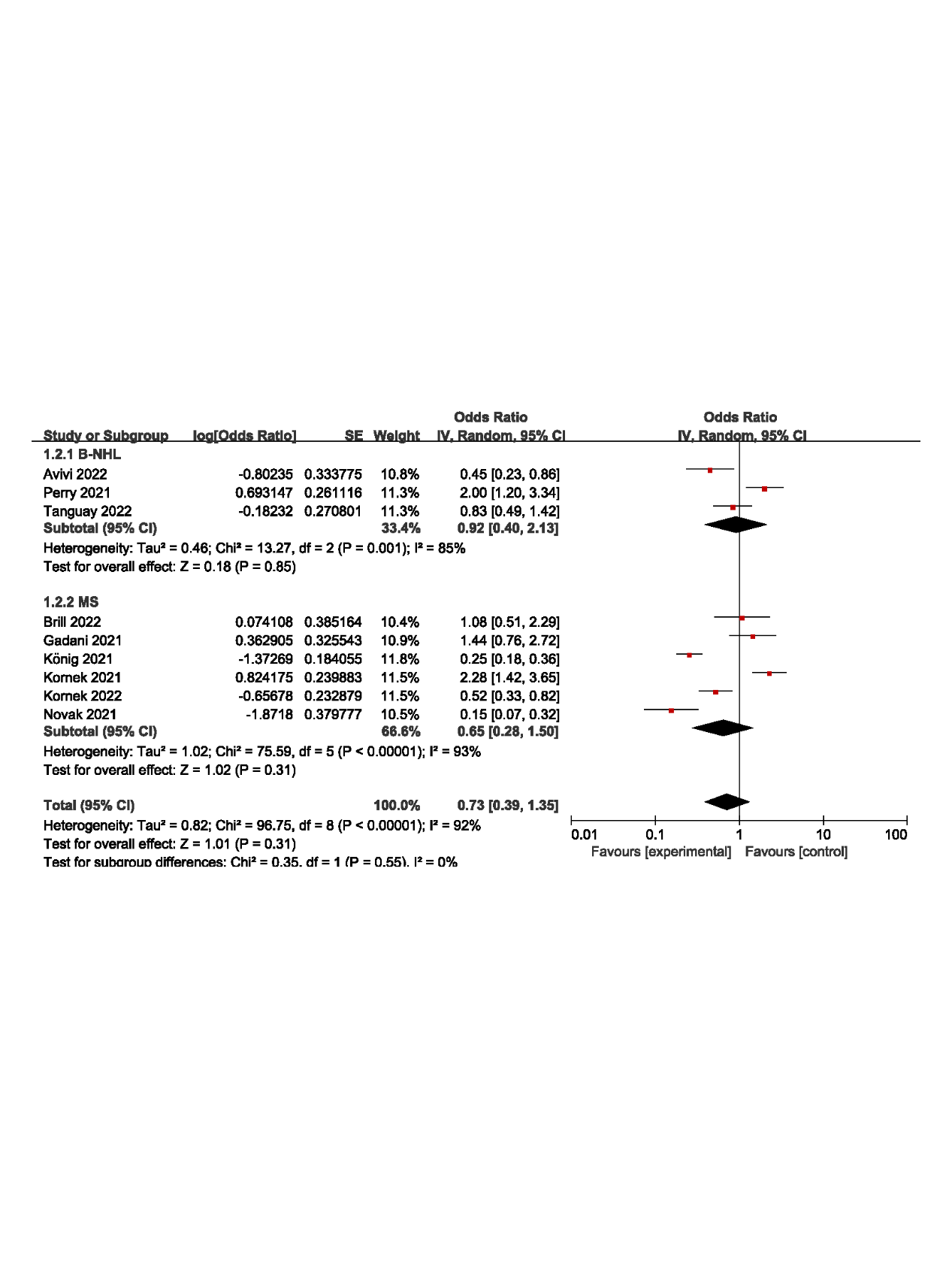


Supplementary Figure 1: Humoral immune responses according to different disease types.

Supplementary Figure 2:
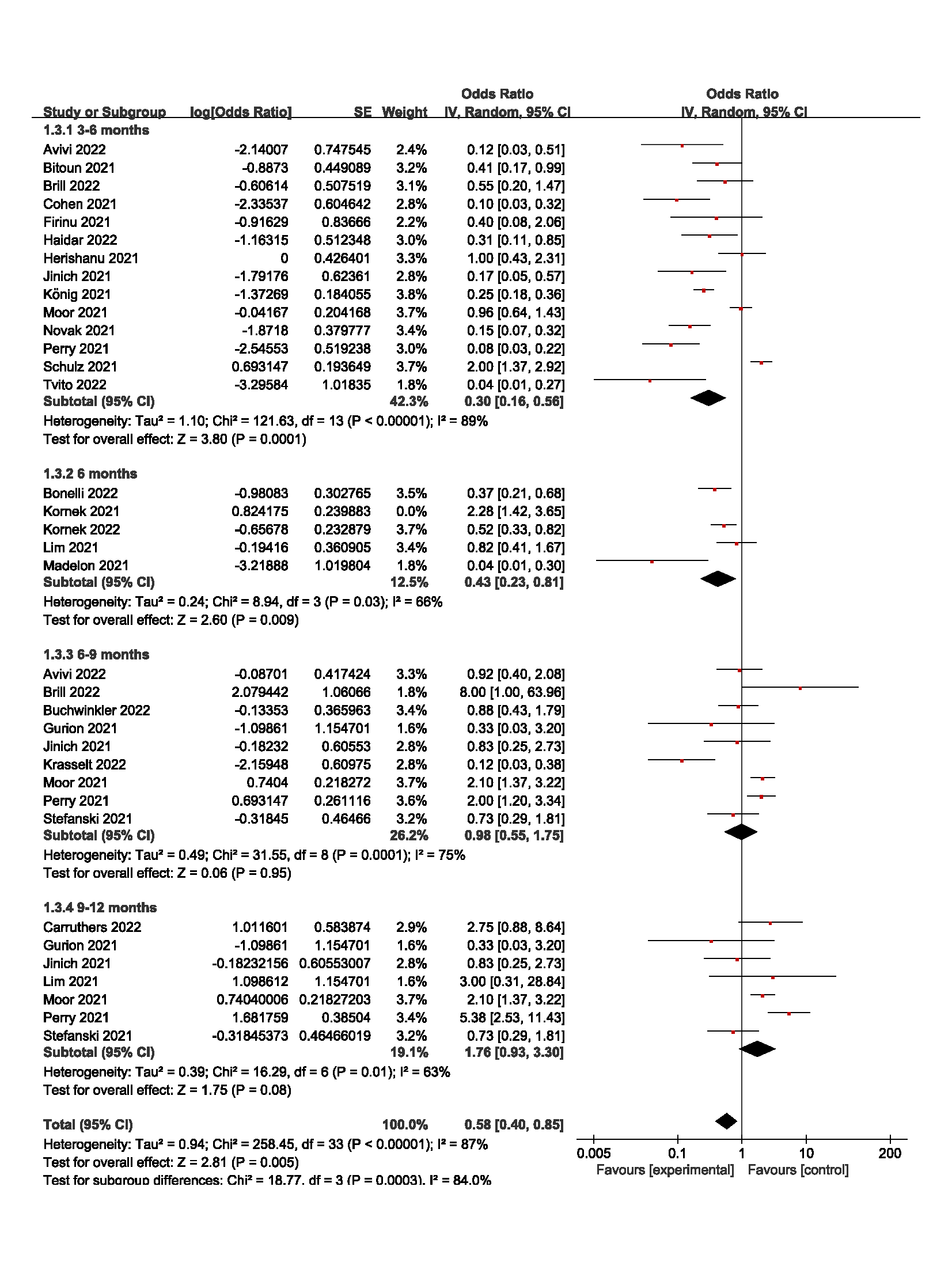
 Humoral immune responses according to prespecified subgroups of 3-6, 6, 6-9 and 9-12 months since the last dose of anti-CD20 therapy after removing the Kornek 2021.

Supplementary Figure
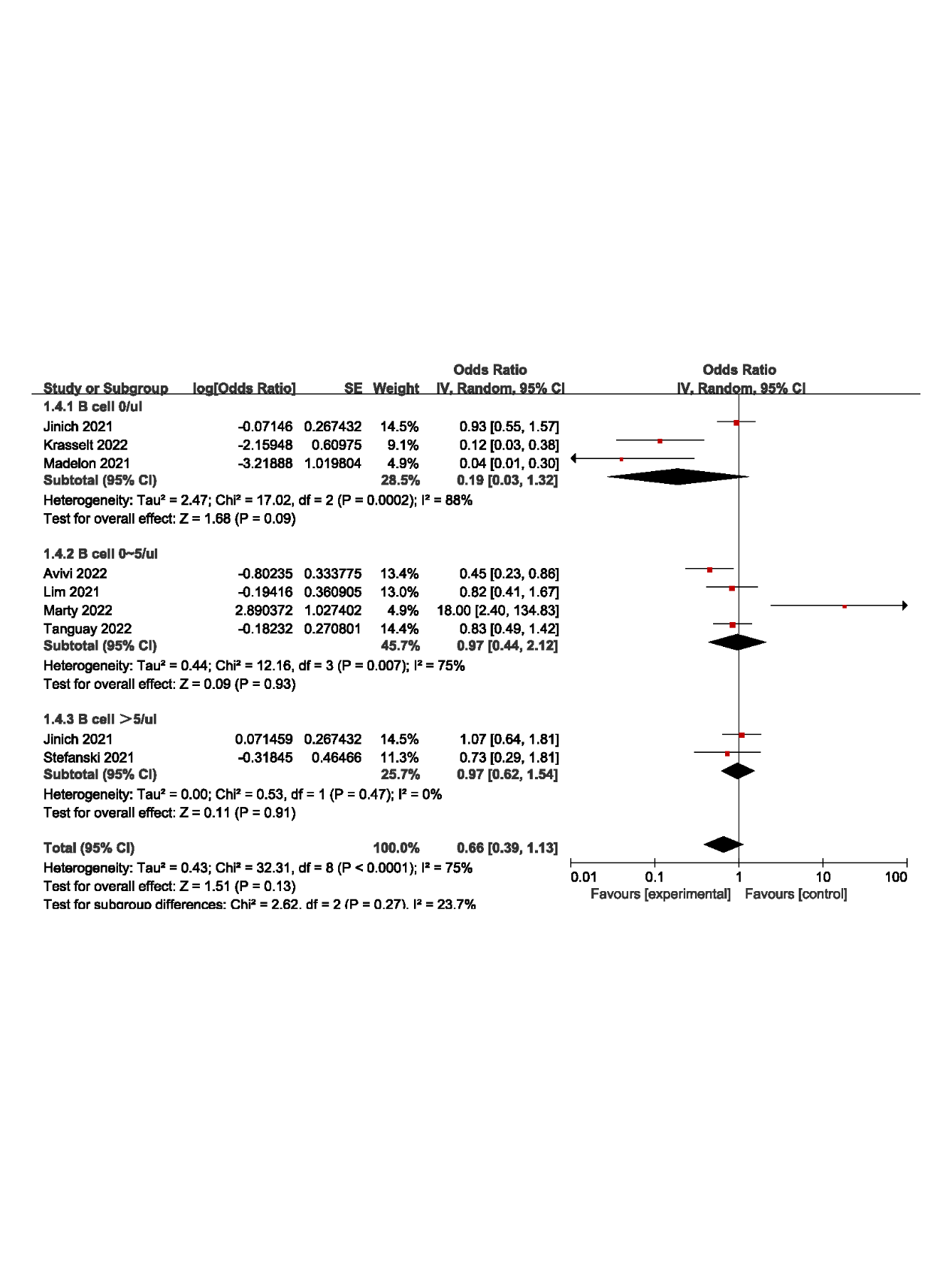
3: Humoral immune responses were stratified by subgroups of patients with depleted B-cell counts, B-cells 0-5ul and B-cells 5ul.

| Supplementary Table 1: Area under the ROC curve | |
| --- | --- |
| The area under the ROC curve |  |
| Area | 0.6863 |
| Std. Error | 0.01395 |
| 95% confidence interval | 0.6589 to 0.7136 |
| P value | <0.0001 |
|  |  |
| Data |  |
| Controls (Negative group) | 872 |
| Patients (Positive group) | 604 |
| Missing Controls | 0 |
| Missing Patients | 0 |

| Supplementary Table 2: Sensitivity, specificity, and Jorden index of ROC curve | | | | | | |
| --- | --- | --- | --- | --- | --- | --- |
|  | Sensitivity | 95% CI | Specificity | 95% CI | Likelihood ratio | Jorden Index |
| > 0.9150 | 0.9983 | 0.9907 to 0.9999 | 0.03211 | 0.02231 to 0.04602 | 1.031 | 0.03041 |
| > 1.250 | 0.9603 | 0.9416 to 0.9732 | 0.05046 | 0.03780 to 0.06706 | 1.011 | 0.01076 |
| > 1.875 | 0.9487 | 0.9281 to 0.9636 | 0.1101 | 0.09100 to 0.1326 | 1.066 | 0.0588 |
| > 2.625 | 0.9404 | 0.9186 to 0.9566 | 0.1284 | 0.1079 to 0.1523 | 1.079 | 0.0688 |
| > 3.250 | 0.9354 | 0.9129 to 0.9524 | 0.164 | 0.1409 to 0.1900 | 1.119 | 0.0994 |
| > 3.750 | 0.9354 | 0.9129 to 0.9524 | 0.1835 | 0.1592 to 0.2105 | 1.146 | 0.1189 |
| > 4.250 | 0.8609 | 0.8310 to 0.8862 | 0.4106 | 0.3784 to 0.4435 | 1.461 | 0.2715 |
| > 4.550 | 0.8593 | 0.8293 to 0.8847 | 0.414 | 0.3817 to 0.4470 | 1.466 | 0.2733 |
| > 4.800 | 0.8593 | 0.8293 to 0.8847 | 0.445 | 0.4123 to 0.4781 | 1.548 | 0.3043 |
| > 5.150 | 0.8179 | 0.7851 to 0.8466 | 0.4725 | 0.4395 to 0.5057 | 1.55 | 0.2904 |
| > 5.400 | 0.8179 | 0.7851 to 0.8466 | 0.5057 | 0.4726 to 0.5388 | 1.655 | 0.3236 |
| > 5.515 | 0.8146 | 0.7816 to 0.8435 | 0.5115 | 0.4783 to 0.5445 | 1.667 | 0.3261 |
| > 5.765 | 0.6838 | 0.6456 to 0.7196 | 0.5573 | 0.5242 to 0.5900 | 1.545 | 0.2411 |
| > 6.500 | 0.4934 | 0.4537 to 0.5332 | 0.742 | 0.7119 to 0.7699 | 1.912 | 0.2354 |
| > 7.150 | 0.4884 | 0.4487 to 0.5282 | 0.7718 | 0.7428 to 0.7984 | 2.14 | 0.2602 |
| > 7.550 | 0.3377 | 0.3012 to 0.3764 | 0.8647 | 0.8404 to 0.8858 | 2.496 | 0.2024 |
| > 7.900 | 0.3262 | 0.2900 to 0.3645 | 0.8647 | 0.8404 to 0.8858 | 2.41 | 0.1909 |
| > 8.500 | 0.303 | 0.2677 to 0.3408 | 0.883 | 0.8600 to 0.9027 | 2.59 | 0.186 |
| > 9.750 | 0.2831 | 0.2486 to 0.3203 | 0.9037 | 0.8823 to 0.9215 | 2.939 | 0.1868 |
| > 11.25 | 0.2616 | 0.2281 to 0.2981 | 0.9037 | 0.8823 to 0.9215 | 2.716 | 0.1653 |
| > 12.42 | 0.2334 | 0.2015 to 0.2688 | 0.9083 | 0.8873 to 0.9257 | 2.545 | 0.1417 |
| > 16.32 | 0.04801 | 0.03364 to 0.06810 | 1 | 0.9956 to 1.000 |  | 0.04801 |

| Supplementary Table 3: List of the screened studies | | | | |
| --- | --- | --- | --- | --- |
| First author | **Included** | **Exclusion stage** | **Exclusion reason** | **DOI** |
| Avivi | Y | NA | - | 10.1111/bjh.18029 |
| Bitoun | Y | NA | - | 10.1002/art.42058. |
| Bonelli | Y | NA | - | 10.1136/annrheumdis-2021-221558 |
| Brill | Y | NA | - | 10.1101/2022.01.26.22269876 |
| Buchwinkler | Y | NA | - | 10.3390/jcm11010148 |
| Carruthers | Y | NA | - | 10.3389/fmed.2021.817845 |
| Cohen | Y | NA | - | 10.1007/s00259-021-05389-x |
| Diefenbach | Y | NA | - | 10.1101/2021.06.02.21257804 |
| Firinu | Y | NA | - | 10.1007/s10238-021-00771-3 |
| Gadani | Y | NA | - | 10.1101/2021.08.23.21262472 |
| Gurion | Y | NA | - | 10.3324/haematol.2021.279216 |
| Haidar | Y | NA | - | 10.1093/cid/ciac103 |
| Herishanu | Y | NA | - | 10.1182/blood.2021011568 |
| Jinich | Y | NA | - | 10.1002/art.42034 |
| König | Y | NA | - | 10.1136/jnnp-2021-327612 |
| Kornek | Y | NA | - | 10.2139/ssrn.3924204 |
| Kornek | Y | NA | - | 10.1002/ana.26309 |
| Krasselt | Y | NA | - | 10.1093/rheumatology/keac089 |
| Lim | Y | NA | - | 10.1101/2021.12.08.21266760 |
| Madelon | Y | NA | - | 10.1093/cid/ciab954 |
| Marty | Y | NA | - | 10.3389/fimmu.2022.834981 |
| Moor | Y | NA | - | 10.1016/s2665-9913(21)00251-4 |
| Novak | Y | NA | - | 10.1016/j.msard.2021.103251 |
| Perry | Y | NA | - | 10.1182/bloodadvances.2021005094 |
| Schulz | Y | NA | - | 10.1101/2021.08.11.21261898 |
| Stefanski | Y | NA | - | 10.1002/art.42060 |
| Tanguay | Y | NA | - | 10.1016/j.vaccine.2022.01.040 |
| Tvito | Y | NA | - | 10.1016/j.exphem.2021.12.396 |
| Arellano-Llamas | N | abstract | Review or guidelines articles | 10.1007/s11912-022-01198-z |
| Bjork | N | abstract | Review or guidelines articles | 10.1016/j.chest.2021.07.446 |
| Cabreira | N | abstract | Review or guidelines articles | 10.3390/vaccines9070773 |
| Costamagna | N | abstract | Review or guidelines articles | 10.1007/s00415-020-10149-2 |
| Coyle | N | abstract | Review or guidelines articles | 10.1007/s12325-021-01761-3 |
| Etemadifar | N | abstract | Review or guidelines articles | 10.1101/2022.02.12.22270883 |
| Fattizzo | N | abstract | Review or guidelines articles | 10.3389/fimmu.2021.791429 |
| Fitzgerald | N | abstract | Review or guidelines articles | 10.1101/2021.02.03.21251069 |
| Galmiche | N | abstract | Review or guidelines articles | 10.1016/j.cmi.2021.09.036 |
| Haidar | N | abstract | Review or guidelines articles | 10.1101/2021.06.28.21259576 |
| Hasseli | N | abstract | Review or guidelines articles | 10.1007/s00296-021-04986-1 |
| Hatami | N | abstract | Review or guidelines articles | 10.1111/dth.15216 |
| Hueso | N | abstract | Review or guidelines articles | 10.1101/2021.12.29.21268525 |
| Inshasi | N | abstract | Review or guidelines articles | 10.1007/s40120-021-00260-5 |
| Jena | N | abstract | Review or guidelines articles | 10.1016/j.autrev.2021.102927 |
| Jena | N | abstract | Review or guidelines articles | 10.1016/j.autrev.2021.102927 |
| Kant | N | abstract | Review or guidelines articles | 10.1016/j.ekir.2021.03.876 |
| Kelly | N | abstract | Review or guidelines articles | 10.1016/j.jneuroim.2021.577599 |
| Kim | N | abstract | Review or guidelines articles | 10.1182/blood-2021-146738 |
| Kroon | N | abstract | Review or guidelines articles | 10.1136/annrheumdis-2021-221575 |
| Lee | N | abstract | Review or guidelines articles | 10.1101/2021.09.28.21264126 |
| Liao | N | abstract | Review or guidelines articles | 10.1101/2021.08.03.21261483 |
| Marinović | N | abstract | Review or guidelines articles | 10.3390/biomedicines9111555 |
| Mason | N | abstract | Review or guidelines articles | 10.1177/09612033211024355 |
| Monin-Aldama | N | abstract | Review or guidelines articles | 10.1101/2021.03.17.21253131 |
| Morawska | N | abstract | Review or guidelines articles | 10.1111/ejh.13722 |
| Notarte | N | abstract | Review or guidelines articles | 10.1101/2021.10.10.21264825 |
| Park | N | abstract | Review or guidelines articles | 10.3346/jkms.2021.36.e95 |
| Prabhahar | N | abstract | Review or guidelines articles | 10.1007/s00296-021-05069-x |
| Prabhahar | N | abstract | Review or guidelines articles | 10.1007/s00296-021-05069-x |
| Rieckmann | N | abstract | Review or guidelines articles | 10.1101/2021.06.22.21259308 |
| Rizk | N | abstract | Review or guidelines articles | 10.1001/jamacardio.2021.3444 |
| Sakuraba | N | abstract | Review or guidelines articles | 10.1053/j.gastro.2021.09.055 |
| Sakuraba | N | abstract | Review or guidelines articles | 10.1186/s13045-022-01233-3 |
| Santosa | N | abstract | Review or guidelines articles | 10.1111/1756-185X.14107 |
| Schietzel | N | abstract | Review or guidelines articles | 10.1136/rmdopen-2021-002036 |
| Schietzel | N | abstract | Review or guidelines articles | 10.1101/2021.09.30.21264335 |
| Sellebjerg | N | abstract | Review or guidelines articles | 10.1097/wco.0000000000000938 |
| Shields | N | abstract | Review or guidelines articles | 10.1182/blood-2021-150220 |
| Sidler | N | abstract | Review or guidelines articles | 10.1101/2021.11.19.21266572 |
| Sidler | N | abstract | Review or guidelines articles | 10.1101/2021.11.19.21266572 |
| Soy | N | abstract | Review or guidelines articles | 10.1007/s10067-021-05700-z |
| Sparks | N | abstract | Review or guidelines articles | 10.1097/bor.0000000000000812 |
| Tang | N | abstract | Review or guidelines articles | 10.1007/s11926-021-01046-2 |
| Teh | N | abstract | Review or guidelines articles | 10.1101/2021.11.06.21265967 |
| Vijenthira | N | abstract | Review or guidelines articles | 10.1182/bloodadvances.2021004629 |
| Waldman | N | abstract | Review or guidelines articles | 10.1016/j.jaad.2020.10.075 |
| Waldman | N | abstract | Review or guidelines articles | 10.1016/j.jaad.2021.08.031 |
| Wallach | N | abstract | Review or guidelines articles | 10.1177/13524585211044647 |
| Windpessl | N | abstract | Review or guidelines articles | 10.1055/a-1550-7288 |
| Wolf | N | abstract | Review or guidelines articles | 10.1212/cpj.0000000000001088 |
| Yarahmadi | N | abstract | Review or guidelines articles | 10.30476/ijms.2021.88717.1946 |
| Yarahmadi | N | abstract | Review or guidelines articles | 10.30476/ijms.2021.88717.1946 |
| Ahn | N | abstract | Off topic | 10.1101/2021.05.26.21257700 |
| Alroughani | N | abstract | Off topic | 10.1177/13524585211047080 |
| Alzaid | N | abstract | Off topic | 10.1101/2020.06.02.20119909 |
| Ansari | N | abstract | Off topic | 10.1101/2021.08.12.21261970 |
| Asashima | N | abstract | Off topic | 10.1101/2021.03.13.21253527 |
| Ayers | N | abstract | Off topic | 10.1182/blood-2021-146165 |
| Azzolini | N | abstract | Off topic | 10.1101/2022.01.21.22269633 |
| Bassi | N | abstract | Off topic | 10.1101/2021.10.05.21264054 |
| Behrens | N | abstract | Off topic | 10.1101/2021.12.25.21268392 |
| Ben | N | abstract | Off topic | 10.1136/annrheumdis-2021-eular.3667 |
| Bock | N | abstract | Off topic | 10.1101/2021.12.22.21268127 |
| Braun-Moscovici | N | abstract | Off topic | 10.1101/2021.04.02.21254493 |
| Bruiners | N | abstract | Off topic | 10.1101/2022.02.03.22269612 |
| Brumme | N | abstract | Off topic | 10.1101/2021.10.03.21264320 |
| Cesaro | N | abstract | Off topic | 10.1101/2022.01.06.22268792 |
| Chen | N | abstract | Off topic | 10.1101/2020.04.06.20055475 |
| de Bruijn | N | abstract | Off topic | 10.1111/jth.15418 |
| Deepak | N | abstract | Off topic | 10.1101/2021.04.05.21254656 |
| Dolton | N | abstract | Off topic | 10.1101/2021.06.21.21259010 |
| Ekşioğlu-Demiralp | N | abstract | Off topic | 10.1101/2020.08.01.20166587 |
| Etemadifar | N | abstract | Off topic | 10.1101/2021.10.17.21265114 |
| Fendler | N | abstract | Off topic | 10.1101/2020.12.21.20248608 |
| Fendler | N | abstract | Off topic | 10.1038/s43018-021-00274-w |
| GeurtsvanKessel | N | abstract | Off topic | 10.1101/2021.12.27.21268416 |
| Goel | N | abstract | Off topic | 10.1101/2021.03.03.21252872 |
| Gonçalves | N | abstract | Off topic | 10.1101/2021.05.03.21256416 |
| Greenberger | N | abstract | Off topic | 10.1182/blood-2021-151419 |
| Helfgott | N | abstract | Off topic | 10.1101/2022.01.26.22269932 |
| Henriquez | N | abstract | Off topic | 10.1101/2021.08.08.21261769 |
| Hoornaert | N | abstract | Off topic | 10.1016/S2152-2650(21)02274-6 |
| Huzly | N | abstract | Off topic | 10.1101/2021.07.17.21260316 |
| Irsara | N | abstract | Off topic | 10.1101/2021.02.17.21251907 |
| Irsara | N | abstract | Off topic | 10.1101/2020.11.27.20239590 |
| Kared | N | abstract | Off topic | 10.1101/2022.01.13.22269213 |
| Katz | N | abstract | Off topic | 10.1016/j.msard.2021.103382 |
| Khatamzas | N | abstract | Off topic | 10.1101/2021.01.10.20248871 |
| Lim | N | abstract | Off topic | 10.1101/2021.06.05.21258311 |
| Machado | N | abstract | Off topic | 10.1136/annrheumdis-2021-eular.5097 |
| Mamez | N | abstract | Off topic | 10.1038/s41409-021-01466-9 |
| Maneikis | N | abstract | Off topic | 10.1016/s2352-3026(21)00169-1 |
| Marcos-Jiménez | N | abstract | Off topic | 10.1101/2020.06.15.20131706 |
| Martínez-Gallo | N | abstract | Off topic | 10.1101/2021.03.31.21254472 |
| Mateen | N | abstract | Off topic | 10.1007/s00415-020-10045-9 |
| Monin-Aldama | N | abstract | Off topic | 10.1101/2021.03.17.21253131 |
| Perkmann | N | abstract | Off topic | 10.1101/2021.08.26.21262426 |
| Perugino | N | abstract | Off topic | 10.1101/2021.12.30.21268554 |
| Phillips | N | abstract | Off topic | 10.1101/2022.02.05.22270447 |
| Qiao | N | abstract | Off topic | 10.1093/milmed/usab314 |
| Re | N | abstract | Off topic | 10.1101/2021.07.18.21260669 |
| Schena | N | abstract | Off topic | 10.1177/03008916211041664 |
| Shen | N | abstract | Off topic | 10.1101/2021.10.28.21265549 |
| Shopen | N | abstract | Off topic | 10.1101/2022.01.29.22270090 |
| Shukla | N | abstract | Off topic | 10.1136/archdischild-2021-rcpch.59 |
| Singh | N | abstract | Off topic | 10.1182/blood-2021-153307 |
| Takahashi | N | abstract | Off topic | 10.1101/2020.06.06.20123414 |
| Terpos | N | abstract | Off topic | 10.1182/blood-2021-144856 |
| Uwamino | N | abstract | Off topic | 10.1101/2021.12.14.21267039 |
| Vaisman-Mentesh | N | abstract | Off topic | 10.1101/2020.08.23.20179796 |
| Verhoeven | N | abstract | Off topic | 10.1016/j.jbspin.2021.105283 |
| Vikkurthi | N | abstract | Off topic | 10.1101/2021.11.14.21266294 |
| Wong | N | abstract | Off topic | 10.1101/2021.03.15.21253615 |
| Zeng | N | abstract | Off topic | 10.1101/2021.10.20.21265273 |
| Zimmerman | N | abstract | Off topic | 10.1101/2022.01.26.22269848 |
| Lucas Bochnia-Bueno | N | abstract | Off topic | NA |
| Lúcio Requião-Moura | N | abstract | Off topic | NA |
| Eric Wolf | N | abstract | Off topic | NA |
| Brogna | N | abstract | n ≤ 3 | 10.3390/medicina57090891 |
| Canti | N | abstract | n ≤ 3 | 10.1186/s13045-021-01190-3 |
| Chilimuri | N | abstract | n ≤ 3 | 10.1093/rap/rkab038 |
| Conte | N | abstract | n ≤ 3 | 10.1177/1352458520974937 |
| Deucher | N | abstract | n ≤ 3 | 10.1002/rth2.12658 |
| Etemadifar | N | abstract | n ≤ 3 | 10.1080/21645515.2021.1928463 |
| Ferguson | N | abstract | n ≤ 3 | 10.1016/j.ijid.2021.06.054 |
| Flannery | N | abstract | n ≤ 3 | 10.3389/fneur.2021.764197 |
| Flower | N | abstract | n ≤ 3 | 10.1136/bcr-2021-245218 |
| Francisco | N | abstract | n ≤ 3 | 10.1182/blood-2021-151964 |
| Gonzalez | N | abstract | n ≤ 3 | 10.1016/j.chest.2021.07.1265 |
| Haroun | N | abstract | n ≤ 3 | 10.1200/JCO.2021.39.15_suppl.e14562 |
| Jasaraj | N | abstract | n ≤ 3 | 10.7759/cureus.16871 |
| Jubber | N | abstract | n ≤ 3 | 10.1111/1756-185X.14200 |
| Khan | N | abstract | n ≤ 3 | 10.1016/j.chest.2021.07.409 |
| Lee | N | abstract | n ≤ 3 | 10.1136/bcr-2021-246049 |
| Leung | N | abstract | n ≤ 3 | 10.1002/ccr3.4517 |
| Liang | N | abstract | n ≤ 3 | NA |
| Lopez | N | abstract | n ≤ 3 | NA |
| Major | N | abstract | n ≤ 3 | 10.1002/jca.21945 |
| Morgenstern-Kaplan | N | abstract | n ≤ 3 | 10.1016/j.anai.2021.08.187 |
| Murali | N | abstract | n ≤ 3 | 10.1177/10781552221075545 |
| Nicolson | N | abstract | n ≤ 3 | 10.1101/2021.08.17.21262138 |
| Osmanodja | N | abstract | n ≤ 3 | 10.1186/s12882-021-02616-3 |
| Sakano | N | abstract | n ≤ 3 | 10.1080/00325481.2022.2037359 |
| Seif | N | abstract | n ≤ 3 | NA |
| Shakoor | N | abstract | n ≤ 3 | 10.1053/j.ajkd.2021.06.016 |
| Subramanian | N | abstract | n ≤ 3 | 10.1016/j.chest.2021.07.472 |
| Tzarnas | N | abstract | n ≤ 3 | 10.1097/01.ccm.0000726612.71471.eb |
| Verhoeven | N | abstract | n ≤ 3 | 10.1016/j.jbspin.2021.105258 |
| Yilmaz | N | abstract | n ≤ 3 | 10.1016/j.htct.2021.10.1057 |
| Yoshida | N | abstract | n ≤ 3 | 10.2169/internalmedicine.8568-21 |
| Bigaut | N | abstract | SARS-CoV2 infection | 10.1016/j.neurol.2021.05.001 |
| Calabrese | N | abstract | SARS-CoV2 infection | 10.1101/2022.02.21.22271289 |
| Cook | N | abstract | SARS-CoV2 infection | 10.1101/2021.08.04.21261618 |
| Drouin | N | abstract | SARS-CoV2 infection | 10.3390/v13071202 |
| Epple | N | abstract | SARS-CoV2 infection | 10.1177/13524585211044667 |
| Jaber | N | abstract | SARS-CoV2 infection | 10.1177/13524585211044647 |
| Kister | N | abstract | SARS-CoV2 infection | 10.1101/2022.01.10.22268752 |
| Klineova | N | abstract | SARS-CoV2 infection | 10.1016/j.msard.2021.103153 |
| Klineova | N | abstract | SARS-CoV2 infection | 10.1177/13524585211044647 |
| Lee | N | abstract | SARS-CoV2 infection | 10.1101/2021.08.25.21262417 |
| Petri | N | abstract | SARS-CoV2 infection | 10.1002/art.41966 |
| Schiavetti | N | abstract | SARS-CoV2 infection | 10.1101/2022.01.22.22269630 |
| Shepherd | N | abstract | SARS-CoV2 infection | 10.1016/j.annonc.2021.08.1550 |
| Sormani | N | abstract | SARS-CoV2 infection | 10.1101/2021.12.23.21268177 |
| Achtnichts | N | duplicate  check | duplicate | 10.3390/vaccines9121470 |
| Ammitzbøll | N | duplicate  check | duplicate | 10.1002/acr2.11299 |
| Apostolidis | N | duplicate  check | duplicate | 10.1101/2021.06.23.21259389 |
| Arellano-Llamas | N | duplicate  check | duplicate | 10.1007/s11912-022-01198-z |
| Arnold | N | duplicate  check | duplicate | 10.1093/rheumatology/keab223 |
| Avivi | N | duplicate  check | duplicate | 10.1111/bjh.18029 |
| Azzolini | N | duplicate  check | duplicate | 10.26508/lsa.202201381 |
| Baker | N | duplicate  check | duplicate | 10.1093/cei/uxab015 |
| Baker | N | duplicate  check | duplicate | 10.1111/cei.13495 |
| Bellesi | N | duplicate  check | duplicate | 10.1080/10428194.2022.2032042 |
| Benjamini | N | duplicate  check | duplicate | 10.3324/haematol.2021.279196 |
| Bergman | N | duplicate  check | duplicate | 10.1016/j.ebiom.2021.103705 |
| Bigaut | N | duplicate  check | duplicate | 10.1016/j.neurol.2021.05.001 |
| Bitoun | N | duplicate  check | duplicate | 10.1002/art.42058 |
| Boekel | N | duplicate  check | duplicate | 10.1016/S2665-9913(21)00222-8 |
| Bonelli | N | duplicate  check | duplicate | 10.1136/annrheumdis2021-221558 |
| Branagan | N | duplicate  check | duplicate | 10.1016/S2152-2650(21)02119-4 |
| Brogna | N | duplicate  check | duplicate | 10.3390/medicina57090891 |
| Brosh-Nissimov | N | duplicate  check | duplicate | 10.1016/j.cmi.2021.06.036 |
| Bruchfeld | N | duplicate  check | duplicate | 10.1093/ndt/gfab174 |
| Buchwinkler | N | duplicate  check | duplicate | 10.3390/jcm11010148 |
| Cabreira | N | duplicate  check | duplicate | 10.3390/vaccines9070773 |
| Carruthers | N | duplicate  check | duplicate | 10.3389/fmed.2021.817845 |
| Chiarucci | N | duplicate  check | duplicate | 10.3389/fonc.2021.737300 |
| Cohen | N | duplicate  check | duplicate | 10.1007/s00259-021-05389-x |
| Coyle | N | duplicate  check | duplicate | 10.1007/s12325-021-01761-3 |
| de Bruijn | N | duplicate  check | duplicate | 10.1111/jth.15418 |
| Delmonte | N | duplicate  check | duplicate | 10.1016/j.jaci.2021.08.016 |
| Deucher | N | duplicate  check | duplicate | 10.1002/rth2.12658 |
| Etemadifar | N | duplicate  check | duplicate | 10.1016/j.msard.2021.103417 |
| Etemadifar | N | duplicate  check | duplicate | 10.1080/21645515.2021.1928463 |
| Fattizzo | N | duplicate  check | duplicate | 10.3389/fimmu.2021.791429 |
| Fendler | N | duplicate  check | duplicate | 10.1038/s43018-021-00274-w |
| Fendler | N | duplicate  check | duplicate | 10.1038/s43018-021-00274-w |
| Ferguson | N | duplicate  check | duplicate | 10.1016/j.ijid.2021.06.054 |
| Ferri | N | duplicate  check | duplicate | 10.1016/j.jaut.2021.102744 |
| Firinu | N | duplicate  check | duplicate | 10.1007/s10238-021-00771-3 |
| Flower | N | duplicate  check | duplicate | 10.1136/bcr-2021-245218 |
| Funakoshi | N | duplicate  check | duplicate | 10.1007/s12185-021-03247-y |
| Furer | N | duplicate  check | duplicate | 10.1136/annrheumdis-2021-eular.5096 |
| Furer | N | duplicate  check | duplicate | 10.1136/annrheumdis-2021-eular.5096 |
| Furlan | N | duplicate  check | duplicate | 10.3389/fimmu.2021.763412 |
| Galleguillos | N | duplicate  check | duplicate | 10.1056/NEJMoa2114583 |
| Galmiche | N | duplicate  check | duplicate | 10.1016/j.cmi.2021.09.036 |
| Gavriatopoulou | N | duplicate  check | duplicate | 10.1182/bloodadvances.2021005444 |
| Gurion | N | duplicate  check | duplicate | 10.3324/haematol.2021.279216 |
| Haidar | N | duplicate  check | duplicate | 10.1093/cid/ciac103/6530582 |
| Haskin | N | duplicate  check | duplicate | 10.1097/TP.0000000000003922 |
| Hasseli | N | duplicate  check | duplicate | 10.1055/a-1616-8742 |
| Hasseli | N | duplicate  check | duplicate | 10.1007/s00296-021-04986-1 |
| Herishanu | N | duplicate  check | duplicate | 10.1182/blood.2021011568 |
| Herishanu | N | duplicate  check | duplicate | 10.1182/blood.2021014085 |
| Herzog Tzarfati | N | duplicate  check | duplicate | 10.1002/ajh.26284 |
| Houot | N | duplicate  check | duplicate | 10.1002/ajh.26284 |
| Inshasi | N | duplicate  check | duplicate | 10.1007/s40120-021-00260-5 |
| Inshasi | N | duplicate  check | duplicate | 10.1007/s40120-021-00260-5 |
| Januel | N | duplicate  check | duplicate | 10.1177/13524585211049737 |
| Jena | N | duplicate  check | duplicate | 10.1016/j.autrev.2021.102927 |
| Jinich | N | duplicate  check | duplicate | 10.1002/art.42034 |
| Jinich | N | duplicate  check | duplicate | 10.1002/art.42034 |
| Kant | N | duplicate  check | duplicate | 10.1016/j.kint.2021.11.012 |
| Kant | N | duplicate  check | duplicate | 10.1016/j.kint.2021.08.020 |
| Kant | N | duplicate  check | duplicate | 10.1016/j.ekir.2021.03.876 |
| Katz | N | duplicate  check | duplicate | 10.1016/j.msard.2021.103382 |
| Kelly | N | duplicate  check | duplicate | 10.1016/j.jneuroim.2021.577599 |
| Klineova | N | duplicate  check | duplicate | 10.1016/j.msard.2021.103153 |
| König | N | duplicate  check | duplicate | 10.1136/jnnp-2021-327612 |
| Kornek | N | duplicate  check | duplicate | 10.1002/ana.26309 |
| Krasselt | N | duplicate  check | duplicate | 10.1093/rheumatology/keac089 |
| Kronbichler | N | duplicate  check | duplicate | 10.1016/j.autrev.2021.102986 |
| Kroon | N | duplicate  check | duplicate | 10.1136/annrheumdis-2021-221575 |
| Madelon | N | duplicate  check | duplicate | 10.1101/2021.12.20.21268128v1 |
| Madelon | N | duplicate  check | duplicate | 10.1093/cid/ciab954 |
| Major | N | duplicate  check | duplicate | 10.21203/rs.3.rs-723623/v1 |
| Maneikis | N | duplicate  check | duplicate | 10.1016/S2352-3026(21)00169-1 |
| Marty | N | duplicate  check | duplicate | 10.3389/fimmu.2022.834981 |
| Mason | N | duplicate  check | duplicate | 10.1177/09612033211024355 |
| Mateen | N | duplicate  check | duplicate | 10.1007/s00415-020-10045-9 |
| Molica | N | duplicate  check | duplicate | 10.1182/blood.2021011568 |
| Moor | N | duplicate  check | duplicate | 10.1016/S2665-9913(21)00251-4 |
| Morawska | N | duplicate  check | duplicate | 10.1111/ejh.13722 |
| Moser | N | duplicate  check | duplicate | 10.1016/j.msard.2022.103560 |
| Murali | N | duplicate  check | duplicate | 10.1177/10781552221075545 |
| Novak | N | duplicate  check | duplicate | 10.1016/j.msard.2021.103251 |
| Osmanodja | N | duplicate  check | duplicate | 10.1186/s12882-021-02616-3 |
| Park | N | duplicate  check | duplicate | 10.3346/jkms.2021.36.e95 |
| Peeters | N | duplicate  check | duplicate | 10.1016/j.esmoop.2021.100274 |
| Perry | N | duplicate  check | duplicate | 10.1182/bloodadvances.2021005094 |
| Pitzalis | N | duplicate  check | duplicate | 10.3389/fimmu.2021.781843 |
| Prabhahar | N | duplicate  check | duplicate | 10.1007/s00296-021-05069-x |
| Qiao | N | duplicate  check | duplicate | 10.1093/milmed/usab314 |
| Rizk | N | duplicate  check | duplicate | 10.1001/jamacardio.2021.3444 |
| Rose | N | duplicate  check | duplicate | 10.1177/20552173211057110 |
| Sabatino | N | duplicate  check | duplicate | 10.1101/2021.09.10.21262933 |
| Sabatino | N | duplicate  check | duplicate | 10.1172/jci.insight.156978 |
| Sakuraba | N | duplicate  check | duplicate | 10.1053/j.gastro.2021.09.055 |
| Sakuraba | N | duplicate  check | duplicate | 10.1186/s13045-022-01233-3 |
| Sakuraba | N | duplicate  check | duplicate | 10.1186/s13045-022-01233-3 |
| Sakuraba | N | duplicate  check | duplicate | 10.1053/j.gastro.2021.09.055 |
| Saleh, M.A. and N.A. Saleh | N | duplicate  check | duplicate | 10.1111/dth.15354 |
| Santosa | N | duplicate  check | duplicate | 10.1101/2021.03.01.21252653 |
| Schietzel | N | duplicate  check | duplicate | 10.1136/rmdopen-2021-002036 |
| Sellebjerg | N | duplicate  check | duplicate | 10.1097/WCO.0000000000000938 |
| Seyahi | N | duplicate  check | duplicate | 10.1007/s00296-021-04910-7 |
| Shakoor | N | duplicate  check | duplicate | 10.1053/j.ajkd.2021.06.016 |
| Sidler | N | duplicate  check | duplicate | 10.1101/2021.11.19.21266572v1 |
| Sormani | N | duplicate  check | duplicate | 10.1101/2021.12.23.21268177v1 |
| Sottini | N | duplicate  check | duplicate | 10.1182/blood-2020-141234 |
| Sparks | N | duplicate  check | duplicate | 10.1097/BOR.0000000000000812 |
| Stefanski | N | duplicate  check | duplicate | 10.1002/art.42060 |
| Stefanski | N | duplicate  check | duplicate | 10.1002/art.42060 |
| Tallantyre | N | duplicate  check | duplicate | 10.1002/ana.26251 |
| Tallantyre | N | duplicate  check | duplicate | 10.1002/ana.26251 |
| Tang | N | duplicate  check | duplicate | 10.1007/s11926-021-01046-2 |
| Terpos | N | duplicate  check | duplicate | 10.1182/bloodadvances.2021005444 |
| Thakkar | N | duplicate  check | duplicate | 10.1016/j.ccell.2021.06.002 |
| Tvito | N | duplicate  check | duplicate | 10.1016/j.exphem.2021.12.396 |
| Vijenthira | N | duplicate  check | duplicate | 10.1182/bloodadvances.2021004629 |
| Waldman | N | duplicate  check | duplicate | 10.1016/j.jaad.2020.10.075 |
| Windpessl | N | duplicate  check | duplicate | 10.1055/a-1550-7288 |
| Yarahmadi | N | duplicate  check | duplicate | 10.30476/IJMS.2021.88717.1946 |
| Yoshida | N | duplicate  check | duplicate | 10.2169/internalmedicine.8568-21 |
| Boekel | N | full text | SARS-CoV-2-infection | 10.1016/s2665-9913(21)00222-8 |
| Chiarucci | N | full text | SARS-CoV-2-infection | 10.3389/fonc.2021.737300 |
| Januel | N | full text | SARS-CoV-2-infection | 10.1177/13524585211049737 |
| Mohanraj | N | full text | SARS-CoV-2-infection | 10.1101/2021.12.03.21267250 |
| Prendecki | N | full text | SARS-CoV-2-infection | 10.1136/annrheumdis-2021-220626 |
| Schwarz | N | full text | SARS-CoV-2-infection | 10.1101/2021.10.11.21264694 |
| Ammitzbøll | N | full text | n of responders with history of anti-CD20 therapy not specified | 10.1002/acr2.11299 |
| Apostolidis | N | full text | n of responders with history of anti-CD21 therapy not specified | 10.1038/s41591-021-01507-2 |
| Benjamini | N | full text | n of responders with history of anti-CD22 therapy not specified | 10.3324/haematol.2021.279196 |
| Arnold | N | full text | Review or guidelines articles | 10.1093/rheumatology/keab223 |
| Baker | N | full text | Review or guidelines articles | 10.1093/cei/uxab015 |
| Baker | N | full text | Review or guidelines articles | 10.1111/cei.13495 |
| Barrière | N | full text | Review or guidelines articles | 10.1016/j.ejca.2021.06.008 |
| Bellesi | N | full text | Review or guidelines articles | 10.1080/10428194.2022.2032042 |
| Bernuzzi | N | full text | Review or guidelines articles | 10.1177/03008916211041664 |
| Branagan | N | full text | Review or guidelines articles | 10.1182/blood-2021-154089 |
| Bruchfeld | N | full text | Review or guidelines articles | 10.1093/ndt/gfab174 |
| Chitnis | N | full text | Review or guidelines articles | 10.1177/13524585211044667 |
| Ciampi | N | full text | Review or guidelines articles | 10.1177/13524585211047080 |
| Colmegna | N | full text | Review or guidelines articles | 10.1002/art.41966 |
| Della Pia | N | full text | Review or guidelines articles | 10.1182/blood-2021-151367 |
| Disanto | N | full text | Review or guidelines articles | 10.1177/13524585211047080 |
| Funakoshi | N | full text | Review or guidelines articles | 10.1182/blood-2021-148981 |
| Furer | N | full text | Review or guidelines articles | 10.1002/art.41966 |
| Furlan | N | full text | Review or guidelines articles | 10.3389/fimmu.2021.763412 |
| Galleguillos | N | full text | Review or guidelines articles | 10.1177/13524585211047080 |
| Galleguillos | N | full text | Review or guidelines articles | 10.1177/13524585211044647 |
| Goksu | N | full text | Review or guidelines articles | 10.1182/blood-2021-152930 |
| Guerra | N | full text | Review or guidelines articles | 10.1182/blood-2021-154090 |
| Haberman | N | full text | Review or guidelines articles | 10.1002/art.41966 |
| Hasseli | N | full text | Review or guidelines articles | 10.1055/a-1616-8742 |
| Houot | N | full text | Review or guidelines articles | 10.1016/j.ejca.2020.06.017 |
| Kant | N | full text | Review or guidelines articles | 10.1016/j.kint.2021.11.012 |
| Kant | N | full text | Review or guidelines articles | NA |
| Kirou | N | full text | Review or guidelines articles | 10.1002/art.41966 |
| Kister | N | full text | Review or guidelines articles | 10.1177/13524585211047080 |
| Kister | N | full text | Review or guidelines articles | 10.1177/13524585211047080 |
| Konig | N | full text | Review or guidelines articles | 10.1177/13524585211044647 |
| Kronbichler | N | full text | Review or guidelines articles | 10.1016/j.autrev.2021.102986 |
| Lam | N | full text | Review or guidelines articles | 10.1002/art.41966 |
| Laster | N | full text | Review or guidelines articles | 10.1002/art.41966 |
| Magliulo | N | full text | Review or guidelines articles | 10.1002/art.41966 |
| Mahadeen | N | full text | Review or guidelines articles | 10.1177/13524585211047080 |
| Maillard | N | full text | Review or guidelines articles | 10.1182/blood-2021-149495 |
| Millan-Pascual | N | full text | Review or guidelines articles | 10.1177/13524585211047080 |
| Minehart | N | full text | Review or guidelines articles | 10.1182/blood-2021-153920 |
| Molica | N | full text | Review or guidelines articles | 10.1182/blood-2021-149104 |
| Molina | N | full text | Review or guidelines articles | 10.1182/blood-2021-149568 |
| Moswela | N | full text | Review or guidelines articles | NA |
| Oreja-Guevara | N | full text | Review or guidelines articles | 10.1177/13524585211044667 |
| Raptis | N | full text | Review or guidelines articles | 10.1002/art.41966 |
| Sabatino | N | full text | Review or guidelines articles | 10.1177/13524585211047080 |
| Satyanarayan | N | full text | Review or guidelines articles | 10.1177/13524585211044667 |
| Schieppati | N | full text | Review or guidelines articles | 10.1182/blood-2021-153606 |
| Sertić | N | full text | Review or guidelines articles | 10.1182/blood-2021-153573 |
| Shree | N | full text | Review or guidelines articles | 10.1182/blood-2021-153901 |
| Shree | N | full text | Review or guidelines articles | 10.1158/2643-3230.Bcd-21-0222 |
| Sottini | N | full text | Review or guidelines articles | 10.1182/blood-2020-141234 |
| Sun | N | full text | Review or guidelines articles | 10.1182/blood-2021-148887 |
| Torgauten | N | full text | Review or guidelines articles | 10.1177/13524585211047080 |
| Wallach | N | full text | Review or guidelines articles | 10.1177/1352458520974937 |
| Weinstock-Guttman | N | full text | Review or guidelines articles | 10.1177/13524585211044647 |
| Wolf | N | full text | Review or guidelines articles | 10.1177/13524585211044647 |
| Bonelli | N | full text | Duplicates | 10.1002/art.41966 |
| Madelon | N | full text | Duplicates | 10.1101/2021.07.21.21260928 |
| Sabatino | N | full text | Duplicates | 10.1101/2021.09.10.21262933 |
| Achtnichts | N | full text | Off topic | 10.3390/vaccines9121470 |
| Shrotri | N | full text | Off topic | 10.1101/2021.05.12.21257102 |
| Bajwa | N | full text | Off topic | 10.1101/2022.01.27.22269944 |
| Baker | N | full text | Off topic | 10.1101/2021.09.26.21264023 |
| Brosh-Nissimov | N | full text | Off topic | 10.1016/j.cmi.2021.06.036 |
| Chbat | N | full text | Off topic | 10.1182/blood-2021-150298 |
| Corradini | N | full text | Off topic | 10.1101/2022.01.12.22269133 |
| Delmonte | N | full text | Off topic | 10.1016/j.jaci.2021.08.016 |
| Etemadifar | N | full text | Off topic | 10.1016/j.msard.2021.103417 |
| Ferri | N | full text | Off topic | 10.1016/j.jaut.2021.102744 |
| Fox | N | full text | Off topic | 10.1101/2021.07.19.21260762 |
| Furer | N | full text | Off topic | 10.1136/annrheumdis-2021-220647 |
| Gallo | N | full text | Off topic | 10.1016/j.jns.2021.117795 |
| Gavriatopoulou | N | full text | Off topic | 10.1182/bloodadvances.2021005444 |
| Hadjadj | N | full text | Off topic | 10.1136/annrheumdis-2021-221508 |
| Haskin | N | full text | Off topic | 10.1097/tp.0000000000003922 |
| Herishanu | N | full text | Off topic | 10.1182/blood.2021014085 |
| Herzog Tzarfati | N | full text | Off topic | 10.1002/ajh.26284 |
| Jyssum | N | full text | Off topic | 10.1016/s2665-9913(21)00394-5 |
| Kant | N | full text | Off topic | 10.1016/j.kint.2021.08.020 |
| König | N | full text | Off topic | 10.1101/2021.10.15.21264977 |
| Kufukihara | N | full text | Off topic | 10.1111/cen3.12679 |
| Lyski | N | full text | Off topic | 10.1101/2021.11.04.21265948 |
| Madelon | N | full text | Off topic | 10.1101/2021.12.20.21268128 |
| Meyer-Arndt | N | full text | Off topic | 10.1101/2022.02.06.22270550 |
| Molica | N | full text | Off topic | 10.1159/000521229 |
| Morel | N | full text | Off topic | 10.1016/j.monrhu.2021.11.002 |
| Moser | N | full text | Off topic | 10.1016/j.msard.2022.103560 |
| Patel | N | full text | Off topic | 10.1101/2021.08.05.21261643 |
| Peeters | N | full text | Off topic | 10.1016/j.esmoop.2021.100274 |
| Rossi | N | full text | Off topic | 10.1182/blood-2021-154539 |
| Rubbert-Roth | N | full text | Off topic | 10.1002/art.41966 |
| Sabatino | N | full text | Off topic | 10.1172/jci.insight.156978 |
| Saleh | N | full text | Off topic | 10.1111/dth.15354 |
| Seyahi | N | full text | Off topic | 10.1007/s00296-021-04910-7 |
| Shapiro | N | full text | Off topic | 10.1016/j.ccell.2021.11.006 |
| Shapiro | N | full text | Off topic | 10.1101/2021.09.13.21263365 |
| Shin | N | full text | Off topic | 10.1101/2021.08.17.21262183 |
| Sidler | N | full text | Off topic | 10.1101/2021.11.19.21266572 |
| Sieiro | N | full text | Off topic | 10.1136/rmdopen-2021-001898 |
| Simon | N | full text | Off topic | 10.1136/annrheumdis-2021-221554 |
| Speer | N | full text | Off topic | 10.1136/annrheumdis-2021-221747 |
| Stein | N | full text | Off topic | 10.1101/2021.11.05.21265911 |
| Tallantyre | N | full text | Off topic | 10.1101/2021.07.31.21261326 |
| Tanaka | N | full text | Off topic | 10.1111/cen3.12680 |
| Terpos | N | full text | Off topic | 10.1182/blood-2021-145110 |
| Thakkar | N | full text | Off topic | 10.1101/2021.05.07.21256824 |
| Wagner | N | full text | Off topic | 10.1101/2021.12.13.21267603 |
| Wolf | N | full text | Off topic | 10.1002/ana.26180 |
| Yang | N | full text | Off topic | 10.1101/2022.01.04.22268750 |
| Ziemssen | N | full text | Off topic | 10.1177/13524585211044647 |
| Marc | N | full text | Off topic | NA |
| Ferri | N | full text | Off topic | 10.1016/j.jaut.2021.102744 |
| Kearns | N | full text | Off topic | NA |
| Spelman | N | full text | Off topic | NA |
| Parry | N | full text | Off topic | 10.2139/ssrn.3845994 |
| Funakoshi | N | full text | n ≤ 3 | 10.1007/s12185-021-03247-y |
| Lyski | N | full text | n ≤ 3 | 10.1101/2021.09.02.21262146 |
| Mado | N | full text | n ≤ 3 | 10.1177/03000605211044378 |
| Rose | N | full text | n ≤ 3 | 10.1177/20552173211057110 |

**Y, Yes. N, No. NA, not available**

**Supplementary table 4:** Risk of bias analysis

| Study | Selection | Comparability | Outcome | Overall Risk of Bias |
| --- | --- | --- | --- | --- |
| Avivi | *** | * | *** | **Low**-Medium |
| Bitoun | **** | * | ** | **Low**-Medium |
| Bonelli | *** | ** | *** | Low |
| Brill | *** | * | *** | **Low**-Medium |
| Buchwinkler | *** | * | *** | **Low**-Medium |
| Carruther | ** | * | ** | Low-**Medium** |
| Cohen | *** | * | ** | **Low**-Medium |
| Diefenbach | *** | * | ** | **Low**-Medium |
| Firinu | **** | * | ** | **Low**-Medium |
| Gadani | *** | * | ** | **Low**-Medium |
| Gurion | *** | * | *** | **Low**-Medium |
| Haidar | ** | * | ** | Low-**Medium** |
| Herishanu | *** | * | ** | **Low**-Medium |
| Jinich | *** | * | ** | **Low**-Medium |
| König | *** | * | * | Low-**Medium** |
| Kornek | *** | ** | ** | **Low**-Medium |
| Krasselt | *** | * | * | Low-**Medium** |
| Madelon | ** |  | ** | Medium |
| Marty | *** | * | ** | **Low**-Medium |
| Lim | *** | * | ** | **Low**-Medium |
| Moor | *** | ** | ** | Low |
| Novak | ** | * | ** | Low-**Medium** |
| Perry | **** | * | ** | **Low**-Medium |
| Schulz | *** | * | ** | **Low**-Medium |
| Kornek | *** | * | ** | **Low**-Medium |
| Stefanski | **** | * | * | Low-**Medium** |
| Tanguay | ** | * | *** | **Low**-Medium |
| Tvito | *** | * | *** | **Low**-Medium |

**Scoring used for Newcastle-Ottawa assessment tool**

| Domain | Good Quality/Low Risk | Fair Quality/Medium Risk | Low Quality/High Risk |
| --- | --- | --- | --- |
| Selection | 3-4 stars | 2 stars | 0-1 stars |
| Comparability | 2 stars | 1 star | 0 stars |
| Outcome | 2-3 stars | 1 star | 0 stars |

For mixed ratings, i.e. “low-medium”, the overall rating is closer to the **bold-marked** rating
